# 2436: Disagreement in middle ear volume values between tympanometry and 3-dimensional volume reconstruction

**DOI:** 10.1017/cts.2017.50

**Published:** 2018-05-10

**Authors:** David Carpenter, Debara L. Tucci, David M. Kaylie, Dennis O. Frank-Ito

## Abstract

OBJECTIVES/SPECIFIC AIMS: Middle ear volume (MEV) is a clinically relevant parameter in the treatment of many common conditions including otitis media, tinnitus, and conductive hearing loss. A growing number of studies have shifted from using tympanometry to 3-dimensional volume reconstruction (3DVR) to calculate MEV; however, MEV values between these methodologies have never before been directly compared. Here, our objective is to characterize agreement between MEV measurement methods across disease states and middle ear sizes. METHODS/STUDY POPULATION: Middle ears were identified from 36 patients ranging 18–89 years of age who underwent tympanometry testing during preoperative workup for tympanic membrane (TM) perforation, up to 1 month prior to a standard-of-care temporal bone computed tomography (CT) between October 15, 2005 and October 15, 2015. MEV values calculated by both tympanometry and 3DVR were analyzed for agreement using Bland and Altman plots. A correction factor was calculated where ear canal volumes were available for contralateral middle ears without TM perforation (n=12), and was applied to a second Bland and Altman plot in the corresponding patient subgroup. MEV agreement was characterized across MEV quartiles (1=smallest; 4=largest) and across increasing states of middle ear disease using Kruskal-Wallis and Wilcoxon testing with Bonferonni correction. RESULTS/ANTICIPATED RESULTS: A Bland Altman plot demonstrated significant disagreement of MEV differences as compared to a priori clinical thresholds. Absolute MEV difference was significantly greater in the average MEV fourth to first quartile (*p*=0.0024), fourth to second quartile (*p*=0.0024), third to first quartile (*p*=0.0048), and third to second quartile (*p*=0.048). Absolute MEV difference was not significantly different across varying states of middle ear disease (*p*=0.44). DISCUSSION/SIGNIFICANCE OF IMPACT: Statistically evident and clinically significant disagreement was demonstrated across tympanometric and 3DVR MEV estimates. This lack of agreement was most pronounced at higher average MEV and was persistent yet not appreciably different across varying severities of middle ear disease. These findings may limit the generalizability of studies of the middle ear that differ in MEV estimation methodology, particularly in pathophysiological states where MEV is increased.

